# Training in statistical analysis reduces the framing effect among medical students and residents in Argentina

**DOI:** 10.3352/jeehp.2020.17.25

**Published:** 2020-09-01

**Authors:** Raúl Alfredo Borracci, Eduardo Benigno Arribalzaga, Jorge Thierer

**Affiliations:** 1 Biostatistics, School of Medicine, Austral University, Buenos Aires, Argentina; 2 School of Medicine, Buenos Aires University, Buenos Aires, Argentina; Hallym University, Korea

**Keywords:** Biostatistics, Biometry, Clinical decision-making, Internship and residency, Medical students, Argentina

## Abstract

**Purpose:**

The framing effect refers to a phenomenon wherein, when the same problem is presented using different representations of information, people make significant changes in their decisions. This study aimed to explore whether the framing effect could be reduced in medical students and residents by teaching them the statistical concepts of effect size, probability, and sampling for use in the medical decision-making process.

**Methods:**

Ninety-five second-year medical students and 100 second-year medical residents of Austral University and Buenos Aires University, Argentina were invited to participate in the study between March and June 2017. A questionnaire was developed to assess the different types of framing effects in medical situations. After an initial administration of the survey, students and residents were taught statistical concepts including effect size, probability, and sampling during 2 individual independent official biostatistics courses. After these interventions, the same questionnaire was randomly administered again, and pre- and post-intervention outcomes were compared among students and residents.

**Results:**

Almost every type of framing effect was reproduced either in the students or in the residents. After teaching medical students and residents the analytical process behind statistical concepts, a significant reduction in sample-size, risky-choice, pseudo-certainty, number-size, attribute, goal, and probabilistic formulation framing effects was observed.

**Conclusion:**

The decision-making of medical students and residents in simulated medical situations may be affected by different frame descriptions, and these framing effects can be partially reduced by training individuals in probability analysis and statistical sampling methods.

## Introduction

### Background/rationale

Errors in clinical reasoning are a consequence of cognitive biases, knowledge deficits, and dual-process thinking. To date, at least 38 cognitive biases have been described, most of which are associated with diagnostic errors [1]. A type of bias known as the framing effect refers to a phenomenon wherein, when the same problem is presented using different representations of information, people make significant changes in their decisions or even reverse their decisions [2]. Six types of framing effects have been described: risky-choice framing, attribute framing, goal framing, pseudo-certainty effect, and sample-size, and number-size framing [3,4]. Risky-choice framing generates more positive evaluations when treatment options are described in terms of survival rates instead of mortality rates [3]. Attribute framing is the positive versus the negative description of the specific attribute of a state, such as the chance of getting better with treatment versus the chance of not getting better with the same treatment. Goal framing is the description of the consequences of performing or not performing an act as a gain versus a loss; for example, “if you undergo a screening test for cancer, your survival will be prolonged” versus “if you don’t undergo a screening test, your survival will be shortened” [4]. Number-size framing reflects that people are, for example, more sensitive to the numerical change from 1 to 2 than to the change from 101 to 102. The pseudo-certainty effect highlights the contrast between reduction and elimination of risk. Finally, the sample-size framing effect appears when individuals fail to appreciate that statistical parameters from smaller samples are more variable than those from larger samples.

Several studies have shown that the framing effect also occurs in the medical field, even among physicians, who are formally trained in medical decision-making [2,3,5]. Strategies directed at reducing the effect of cognitive biases can be designed to educate participants about possible biases, with the assumption that this awareness will reduce diagnostic errors [6,7]. Only a few studies have examined the effects of educational interventions designed to teach participants to recognize specific cognitive biases in diagnostic reasoning [8-10]. In particular, most medical students have difficulty in reasoning about chance events and maintain misconceptions regarding probability [11]. Other investigators have explored how a low ability to understand and use numerical information distorts risk and benefit perceptions, both in members of the general public and among doctors [12]. However, recent research reported that it is possible to reduce some types of framing effects in adults by encouraging analytical processing and teaching them “to think like a scientist” to solve experimental decision trials [13].

In addition to statistics, an understanding of probability is essential to informed decision-making in medicine; however, many biostatistics classes in medical schools worldwide prioritize teaching exploratory data analysis over probability [11]. Probability is a rather counterintuitive idea, and since most types of framing effects are related to the concepts of probability, chance, and sampling size, framing effects may largely arise from a knowledge deficit in these topics.

### Objectives

Based on this theoretical framework, our principal assumption was that some medical errors are a consequence of framing biases, which could be reduced via instruction. The purpose of this study was to explore whether the framing effect could be reduced in medical students and residents by teaching them the statistical concepts of effect size, probability, and sampling for use in the medical decision-making process.

## Methods

### Ethics statement

Participants were assured that the completed questionnaire would be anonymous and confidential. After being informed of the purpose of the study, respondents participated in the survey and expressed their consent by completing the corresponding form. The heads of the medical training institutions provided access to the student population after ethical approval of the protocol (IRB 26(1)-201603).

### Study design

This was a survey-based single-group pre- and post-test interventional study.

### Setting/participants

Between March and June 2017, a prospective, quantitative, experimental study with a single-group pre-post-test design was conducted at 2 schools of medicine of Argentina: Austral University and Buenos Aires University. A total of 95 second-year medical students and 100 second-year medical residents were included in the study using a convenience sampling strategy. The total number of students and residents were recruited from 2 mandatory official biostatistics courses, which lasted a semester for medical students (undergraduate course), and 2 months for residents (postgraduate course). The study design included an initial administration of the survey to assess the presence of the framing effect. After the initial survey, students and residents were taught statistical concepts including effect size, probability, and sampling during the 2 independent official biostatistics courses. No direct reference to the administered baseline questionnaire was made during the courses. After these interventions, the same questionnaire was randomly administered again, and pre- and post-intervention outcomes were separately compared for students and residents.

### Data source/measurement

**Fig. 1.**
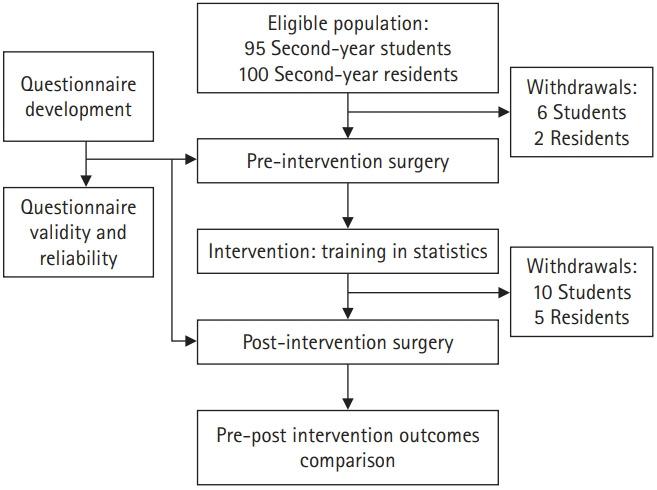
Diagram summarizing the study design.

The administered questionnaire was developed using previously explored questions to assess the different types of framing effects in medical situations [3,14]. Eight representative questions with 15 alternative formulations were selected and randomly included in the questionnaire. The first 2 questions, which assessed the sample-size and risky-choice framing effects, were answered through a dichotomous or trichotomous choice selection. The rest of the questions, which assessed the attribute, goal, pseudo-certainty, probabilistic formulation, and number-size framing effects, had responses on a 6-point Likert scale. The questionnaire structure and the corresponding Likert scale categories are shown in Supplement 1. Questions 1 and 2 were evaluated in the population as a whole; whereas paired formulations of the remaining questions were assessed in 2 independent groups by randomly dividing the total population. The questionnaire was self-administered in hard copy and the data obtained were anonymously included and processed in a database. A summary of the study design is shown in Fig. 1.

### Statistical methods

Categorical data were expressed as absolute frequencies and percentages. Univariate comparison of dichotomous variables was performed using the chi-square test or the 2-tailed Fisher exact test, as appropriate. In these cases, expected observations were calculated by assigning an equal probability to each category of questions 1 and 2. Due to the questionnaire structure, the ceiling effect was expected to occur, and the non-Gaussian distribution of scores was assessed with the Kolmogorov-Smirnov goodness-of-fit test. The median score and the interquartile range (in percentiles) were used as representative values; nevertheless, the mean and standard deviation (SD) were calculated for effect size assessment. The 2-tailed Mann-Whitney U non-parametric test was used to compare non-normal score distributions. The odds ratio (OR), with 95% confidence intervals (CIs), was utilized to assess the effect size for questions with categorical answers. Trichotomous responses for question 1 were collapsed to dichotomous (right/wrong) answers, and independent pre- and post-intervention odds were calculated. The effect size for questions with Likert-scale responses was assessed with the Cohen d index and its derived r coefficient. The sample size was calculated with a power of 0.80 and a significance level of 0.05 based on expected differences in the Likert-scale responses, assuming an SD of 2.5 and a difference to be detected equal to 1.5. The calculated sample size consisted of a total of 87 subjects, individually for student and resident cohorts. Principal component analysis (PCA) and confirmatory factor analysis (CFA) were used to explore construct validity. For PCA, only factors with eigenvalues greater than 1.2 were retained, and factor coefficients greater than 0.40 were required for the interpretation of factor structure, using varimax rotation. The PCA criteria for identifying factor structure were examined using the Kaiser-Meyer-Olkin (KMO) test. To establish whether the data set was suitable for factor analysis, a KMO index greater than 0.50 was required. LISREL ver. 9.20 (Scientific Software International Inc., Skokie, IL, USA) software was used to test the 2-factor structure of the questionnaire by CFA, which investigates how the data fit into a predetermined and constructed model by presenting the relationship between model data and estimated errors. Assessment of model-data fit was done using model chi-square goodness-of-fit and approximate fit indices. A nonsignificant chi-square test (P>0.05) indicates model fit. The additional approximate fit indices employed included the goodness-of-fit index (GFI), the adjusted GFI (AGFI), the normed fit index (NFI), the non-NFI (Tucker–Lewis index; NNFI), the relative fit index (RFI), the incremental fit index (IFI), and the comparative fit index (CFI). Values >0.9 arising from the GFI, AGFI, NFI, NNFI, RFI, IFI, and CFI indicate model fit; conversely, values ≥0.85 represent acceptable model fit. Other indices calculated were the root mean square error of approximation (RMSEA) and the root mean square residual (RMR), in which values <0.08 indicate a reasonable model fit. The internal consistency of the questionnaire was assessed with Cronbach α coefficients based on standardized items and inter-item correlation. An α value greater than 0.60 was considered to indicate acceptable reliability. For validity and reliability analyses, questions 1 and 2 were excluded from the questionnaire since their responses were not based on a Likert scale. Except for CFA, all statistical analyses were performed using SPSS Statistics for Windows ver. 17.0 (SPSS Inc., Chicago, IL, USA), and a P-value less than 0.05 was considered to indicate statistical significance.

## Results

The raw data of responses from 79 students and 93 residents to the questionnaires are presented in Dataset 1.

### Student cohort outcomes

Among 95 eligible medical students, 79 (83.2%) completed the questionnaire with no missing data. The baseline pre-intervention framing effect outcomes related to sample-size (question 1) and risky-choice for candidate equivalent options (question 2) observed in the medical student cohort are shown in the first column of Table 1. The other types of framing effects in the same population are presented in Table 2. The initial administration of the questionnaire to medical students showed a significant occurrence of the sample-size, risky-choice, pseudo-certainty, number-size, probabilistic formulation, and attribute framing effect types. Only the goal framing effect and the risky-choice framing effect presented with non-equivalent options (question 3), were not observed in the pre-intervention assessment. The pre-intervention effect size for each type of framing effect in the medical student cohort is shown in the upper half of Table 3; for methodological reasons, it includes only questions with responses on a Likert scale. The effect size quantification using the Cohen d index demonstrated that the number-size framing effect had a large effect size, whereas the pseudo-certainty, attribute, and probabilistic formulation effects had a medium effect size. The risky-choice and goal framing effects did not have significant effect sizes (see P-values in Table 2). As a measure of effect size, the OR associated with the risky-choice (equivalent options) effect was 1.84 (95% CI, 0.93–3.65), while the OR related to the sample-size framing effect was 3.65.

After the intervention, a significant reduction in the sample-size, risky-choice for equivalent options, and probabilistic formulation framing effects were observed (first 2 columns of Table 1 and Table 2). The pseudo-certainty, number-size, and attribute effects were only partially reduced. As in the baseline assessment, neither the goal framing effect nor the risky-choice effect for non-equivalent options appeared after the intervention. Table 3 presents the magnitude of effect size reduction; excluding the risky-choice effect, all Cohen d indices decreased in the post-intervention evaluation, though the number-size framing effect still had a rather large effect size. Furthermore, the effect size associated with the risky-choice effect with equivalent options was reduced to an OR of 0.85 (95% CI, 0.44–1.63), and the OR related to the sample-size framing effect decreased to 0.65.

### Resident cohort outcomes

Among the 100 eligible medical residents, 93 (93.0%) completed the questionnaire with no missing data. Baseline pre-intervention framing effect outcomes related to the sample-size (question 1) and risky-choice for candidate equivalent options (question 2) observed in the medical resident cohort are shown in the third column of Table 1. The other types of framing effects in the same population are presented in Table 2. The initial administration of the questionnaire to medical residents showed a significant occurrence of the sample-size, goal, number-size, and attribute framing effect types. On the contrary, the risky-choice, pseudo-certainty, and probabilistic formulation framing effects were not observed in the pre-intervention assessment. The pre-intervention effect size for each type of framing effect in the medical resident cohort is shown in the bottom half of Table 3; for methodological reasons, it includes only questions with responses on the Likert scale. The effect size quantification using the Cohen d index demonstrated that the number-size framing effect had a large effect size, while the goal and attribute framing effects had a medium effect size. The risky-choice, pseudo-certainty, and probabilistic formulation framing effects did not have significant effect sizes (see P-values in Table 2). The effect size associated with the risky-choice effect with equivalent options was shown by an OR of 1.67 (95% CI, 0.91–3.07), while the OR related to the sample-size framing effect was 5.13.

After the intervention, a significant reduction in the goal framing effect was observed (third and fourth columns of Table 1 and Table 2). The sample-size and number-size effects were only partially reduced, and conversely, the attribute framing effect was not reversed after the intervention. As in the baseline assessment, the risky-choice, pseudo-certainty, and probabilistic formulation framing effects did not appear after the intervention. Table 3 presents the magnitude of the effect-size reduction; all significant Cohen d indices decreased in the post-intervention evaluation, except for the attribute framing effect, the effect-size index of which increased. Furthermore, the effect size associated with the risky-choice effect with equivalent options remained unchanged, with an OR of 1.64 (95% CI, 0.89–3.01), and the OR related to the sample-size framing effect decreased to 2.83.

### Psychometric characteristics of the questionnaire

The KMO measure of sampling adequacy was 0.85 and the Bartlett test of sphericity was significant (χ²=580, P=0.000), indicating that the data set was suitable for factor analysis. The 12-item PCA yielded a 2-factor model that accounted for 84.8% of the variance (Table 4). CFA was also conducted to determine the construct validity of the questionnaire. An adequate goodness-of-fit statistic was indicated by a nonsignificant maximum likelihood ratio chi-square (P=0.990). An adequate fit was also observed for the following indices: GFI (0.991), AGFI (0.987), NFI (0.990), RFI (0.988), CFI (1.000), all with values >0.90. The RMSEA (0.001) and RMR (0.031) also indicated a suitable fit. The Cronbach α coefficient based on standardized items was 0.61. The absolute values of inter-item correlations ranged from 0.447 to 0.837, with a mean value of 0.711.

## Discussion

### Key results

Medical students and residents showed different types of framing effects after answering a questionnaire that presented pairs of equivalent simulated clinical situations. After training individuals in probability analysis and sampling methods, some types of framing effects were reduced.

### Interpretation

In the present study, almost every type of framing effect was reproduced either in the students or in the resident population before the intervention. However, teaching medical students and residents the analytical process behind statistical notions seemed to have encouraged critical thinking to solve the simulated medical situations presented in the questionnaire. Insensitivity to sample size was explored with question 1, and the observed framing effect was significantly reduced after training students in statistical sampling methods and confidence boundary principles. In this case, training the residents only achieved a partial reduction of the sample-size framing effect. The pre-intervention outcomes for question 2 (risky-choice with equivalent options) replicated the typical finding of risk aversion with positive frames, and risk-seeking with negative frames. After learning probability concepts, students reversed this effect. Although the risky-choice framing effect with candidate equivalent options was not significant in medical residents, the rather high pre-intervention odds ratio suggested that some degree of effect size remained after the intervention. The lack of the framing effect associated with the risky-choice effect presented with non-equivalent options is harder to explain. Based on the complex formulation of question 3, the possibility of construction bias should not be discarded. The probabilistic protection of a hypothetical vaccine was contrasted with a pseudo-certainty-based formulation of its protective effect. In the student cohort, the pseudo-certainty framing effect observed in question 4 highlighted the contrast between reduction and elimination of risk. Although the pseudo-certainty framing effect was reduced, it maintained a moderate effect size after the intervention in medical students. Since the medical students may have incorporated the basic principles of primary prevention, the formulation of question 5 in terms of reducing or increasing the possibility of suffering from a disease might have resulted in an indistinguishable outcome. This may explain the non-occurrence of the goal framing effect. Nevertheless, the medical residents clearly showed the goal framing effect, which reversed after the intervention. The number-size formulation had the highest effect size among all types of framing effects explored, and it was difficult to eliminate despite the intervention, both for students and medical residents. Question 8 confronted the effect of presenting data based on probabilities or as raw data. Again, this framing effect appeared in the baseline survey of medical students and decreased after the intervention.

### Limitations

First, medical residents were not asked about their previous formal training in statistics, so this factor could not be considered during the analysis of data. Notwithstanding, undergraduate statistics programs in most local schools of medicine are absent, or are at best limited to basic concepts. Consequently, some relevant topics on probability, effect size, and sampling potentially useful for interpreting the different types of framing effects are rarely addressed. Second, some inconsistencies were found between the student and resident outcomes; however, all types of framing effects were reproduced in one or the other cohort, and at least, a partial reversal after the intervention was obtained in every case, except for the attribute framing effect in the residents’ cohort. Third, since the courses given to students and residents were different in length, a possible bias favoring medical student post-intervention performance may be expected. The incorporation of an additional control group that received no training in statistics (a true control pre-post-test design) would have been an alternative design that might have provided more solid evidence [15]. Since anonymized data were collected, no paired pre-post-test analysis was done; and consequently, there was no possibility to account for dependency in the data. Since both medical students and residents were in their second year, it should be considered that sometimes, younger students and residents do not make decisions by themselves, but have to follow a teacher or senior resident or staff member; hence, subject selection could have been biased.

### Comparison with previous studies

Many studies have suggested that framing effect is a widespread and robust phenomenon that regularly appears in various fields of decision-making problems, such as the economy, life-saving decisions, resource allocation, management, medicine, and even daily life. Some investigations have indicated that the framing effect varies according to gender roles in different task domains [16]. Additionally, younger adults are more likely than older ones to choose the risky option for negatively framed high-amount mortality-based decision scenarios [17]. Furthermore, the framing effect can be influenced by pleasant or unpleasant feelings [18], sleep deprivation [19], “big 5” personality traits, and even genetic factors [20]. For instance, individuals homozygous for the short allele at the serotonin transporter gene-linked polymorphic region (5-HTTLPR) and Met allele of COMT Val158Met polymorphism (rs4680) carriers have been described to be more sensitive to framing. Since a pre-post-test analysis was done on the same individuals, these previously mentioned potential confounders were theoretically neutralized in the current study.

### Conclusions

The present study suggests that the decision-making of medical students and residents in simulated medical situations may be affected by different frame descriptions and that some types of framing effects can be partially reduced by training individuals in probability analysis and statistical sampling methods. After the intervention, significant to modest reductions were obtained for the sample-size, risky-choice, goal, attribute, pseudo-certainty, number-size, and probabilistic formulation effects, either in medical students or in-training young physicians. Nevertheless, the attribute and number-size framing effects may be the most difficult cognitive biases to eliminate. Training in statistical methods and probability seems to be a useful tool to preclude or reduce some types of framing effects in medical decision-making.

## Figures and Tables

**Table 1.** Framing effect outcomes of questions 1 and 2 after initial and final administrations of the questionnaire to the medical student cohort and the medical resident cohort

Question	Students				Residents			
	Pre-intervention		Post-intervention		Pre-intervention		Post-intervention	
	No. (%)	P-value	No. (%)	P-value	No. (%)	P-value	No. (%)	P-value
Question 1. Which size hospital reported more babies’ births? (sample-size framing effect)								
Responses:								
(a) A larger hospital	19 (24)		9 (11)		13 (14)		23 (25)	
(b) A smaller hospital^a)^	17 (22)		48 (61)		15 (16)		24 (26)	
(c) About the same (that is, within 5% of each other)	43 (54)	0.0004	22 (28)	<0.0001	64 (70)	0.0001	46 (49)	0.082
Question 2. Which program is better to combat H1N1? (risky-choice framing effect; equivalent options)								
Responses: (positive)								
(a) If program A is adopted, 200 people will be saved.	52 (66)		43 (54)		52 (56)		43 (46)	
(b) If program B is adopted, there is 1/3 probability that 600 people will be saved, and 2/3 probability that no people will be saved.	21 (27)	0.0003	32 (41)	0.413	33 (35)	0.164	44 (48)	0.879
Observations: students considering both options to be equivalent^b)^	6 (7)		4 (5)		8 (9)		6 (6)	
Responses: (negative)								
(a) If program C is adopted, 400 people will die.	43 (54)		46 (58)		36 (39)		33 (35)	
(b) If program D is adopted, there is 1/3 probability that nobody will die, and 2/3 probability that 600 people will die.	32 (41)	0.204	29 (37)	0.147	45 (48)	0.529	55 (60)	0.095
Observations: students considering both options to be equivalent^b)^	4 (5)		4 (5)		12 (13)		5 (5)	

a) Correct answer.

b) Since options (a) and (b) in question 2 are equivalent, we added an additional open choice (observations) where respondents could indicate that they considered both options were equivalent.

**Table 2.** Framing effect outcomes of questions 3 to 9 after initial and final administrations of the questionnaire to the medical student and resident cohorts (2 independent groups)

Question	Students				Residents			
	Pre-intervention		Post-intervention		Pre-intervention		Post-intervention	
	Median (IQR)	P-value	Median (IQR)	P-value	Median (IQR)	P-value	Median (IQR)	P-value
Question 3. Which treatment is better to treat cancer? (risky-choice framing effect; non-equivalent options)								
Responses: (positive)	5 (4–6)		4 (2–5)		5 (2–6)		4.5 (2.8–6)	
Responses: (negative)	5 (4–6)	0.909	5 (2–6)	0.085	5 (2–6)	0.975	4 (2.8–5)	0.340
Question 4. Which vaccine is better to protect the population? (pseudo-certainty framing effect)								
Responses: (probabilistic certainty)	5 (3–6)		5 (2–6)		5 (3.5–6)		4.5 (4–6)	
Responses: (pseudocertainty)	6 (5–6)	0.008	6 (5–6)	0.045	6 (5–6)	0.194	5 (3–6)	0.992
Question 5. Diet to reduce cardiovascular risk (goal framing effect)								
Responses: (positive)	1 (1–2)		1 (1–2)		2 (1–3.8)		1 (1–2)	
Responses: (negative)	1 (1–2)	0.361	1 (1–2)	0.934	1 (1–2)	0.005	2 (1–3)	0.098
Question 6. Whether to prefer potential sequelae after eye surgery (number-size framing effect)								
Responses: (framing 1)	3 (1–4)		2 (1–4)		3 (2–5)		2.5 (1.8–5)	
Responses: (framing 2)	5 (3–6)	<0.0001	4 (2–5)	0.003	6 (5–6)	<0.0001	5 (3–6)	0.005
Question 7. How to evaluate a drug effect (attribute framing effect)								
Responses: (positive)	4 (4–5)		4 (4–4.5)		5 (4–5)		5 (4–5)	
Responses: (negative)	4 (4–4)	0.006	4 (3–4)	0.007	4 (3–5)	0.005	4 (3–5)	<0.0001
Question 8. Which vaccine is better for children? (probabilistic formulation framing effect)								
Responses: (probability)	5 (4–5)		5 (5–6)		5.5 (5–6)		5 (4–6)	
Responses: (raw data)	5 (5–6)	0.032	5 (4–6)	0.114	6 (4–6)	0.805	5 (3–6)	0.086

IQR, interquartile range.

**Table 3.** Effect size indexes of each type of framing effect for the medical student and resident cohorts

Cohort	Category	Risky-choice	Pseudo-certainty	Goal	Number-size	Attribute	Probabilistic
Student cohort	Pre-intervention						
	Cohen d	0.01	0.66	0.28	0.87	0.65	0.45
	r coefficient	0.01	0.31	0.14	0.40	0.31	0.22
	Post-intervention						
	Cohen d	0.29	0.55	0.02	0.70	0.55	0.40
	r coefficient	0.15	0.27	0.01	0.33	0.26	0.20
Resident cohort	Pre-intervention						
	Cohen d	0.04	0.28	0.69	0.98	0.71	0.03
	r coefficient	0.02	0.14	0.33	0.44	0.33	0.02
	Post-intervention						
	Cohen d	0.21	0.00	0.35	0.62	1.03	0.36
	r coefficient	0.11	0.00	0.17	0.30	0.46	0.18

**Table 4.** Principal component analysis of items in the framing effect questionnaire

Item	Factor 1	Factor 2	Communalities
Question 3 (positive)	0.752	-	0.70
Question 3 (negative)	-	0.919	0.88
Question 4 (probabilistic certainty)	0.735	-	0.96
Question 4 (pseudo-certainty)	0.767	-	0.84
Question 5 (positive)	-0.887	-	0.98
Question 5 (negative)	0.836	-	0.90
Question 6 (framing 1)	0.799	-	0.78
Question 6 (framing 2)	0.844	-	0.72
Question 7 (positive)	-0.663	-	0.87
Question 7 (negative)	-	-0.910	0.89
Question 8 (probability)	0.709	-	0.91
Question 8 (raw data)	0.790	-	0.76
Eigenvalues	8.97	1.21	
% of variance	74.7	10.1	

Rotation method: varimax with Kaiser normalization. Questions 1 and 2 were excluded from the analysis since their responses were not based on a Likert scale.

## Footnotes

**Data availability** Data files are available from Harvard Dataverse: https://doi.org/10.7910/DVN/BBDHHJ Dataset 1. Raw data of responses from 79 students and 93 residents to the questionnaires.

## Supplementary materials

Data files are available from Harvard Dataverse: https://doi.org/10.7910/DVN/BBDHHJ

Supplement 1. Questionnaire structure to assess the different types of the framing effect.

Supplement 2. Audio recording of the abstract.
