# Supplementary material for: Training in statistical analysis reduces the framing effect among medical students and residents in Argentina
Source: J Educ Eval Health Prof. 2020 Sep 1;17:25. doi: 10.3352/jeehp.2020.17.25 (PMC7577882; doi:10.3352/jeehp.2020.17.25)
Supplement: Supplementary file 2 — Supplement 1. Questionnaire structure to assess the different types of the framing effect. [file jeehp-17-25-suppl1.docx]

**Supplement 1.** Questionnaire structure to assess the different types of framing effect

**Question 1.** Sample-size framing effect (insensitivity to sample size)

A certain town is served by two hospitals. In the larger hospital about 45 babies are born each day, and in the small hospital about 15 babies are born each day. As you know, about 50% of all babies are boys. However, the exact percentage varies from day to day. Sometimes it may be higher than 50%, sometimes lower. For a period of 1 year, each hospital recorded the days on which more than 60% of the babies born were boys. Which hospital do you think recorded more such days? a) The larger hospital; b) The smaller hospital; c) About the same (that is, within 5% of each other).

**Question 2.** Risky-choice framing effect (equivalent options)

*Positive:* Imagine that your country is preparing for the outbreak of H1N1 influenza, which is expected to kill 600 people. Two alternative programs to combat the disease have been proposed. Assume that the exact scientific estimate of the consequences of the programs are as follows: a) If Program A is adopted, 200 people will be saved; b) If Program B is adopted, there is 1/3 probability that 600 people will be saved, and 2/3 probability that no people will be saved. Which of the two programs would you favor?

*Negative:* Imagine that your country is preparing for the outbreak of H1N1 influenza, which is expected to kill 600 people. Two alternative programs to combat the disease have been proposed. Assume that the exact scientific estimate of the consequences of the programs are as follows: a) If Program C is adopted 400 people will die; b) If Program D is adopted there is 1/3 probability that nobody will die, and 2/3 probability that 600 people will die. Which of the two programs would you favor?

**Question 3.** Risky-choice framing effect (not equivalent options)^a^

*Positive:* Imagine one patient was diagnosed with a cancer that must be treated. His choices are as follows: a) Surgery: of 100 people having surgery, 50 live through the operation, and 40 are alive at the end of five years, b) Radiation therapy: of 100 people having radiation therapy, all live through the treatment, and 20 are alive at the end of five years. Which treatment would you advise him to choose?

*Negative:* Imagine one patient was diagnosed with a cancer that must be treated. His choices are as follows: a) Surgery: of 100 people having surgery, 50 die because of the operation and 10 of the 50 survivals die by the end of five years; b) Radiation therapy: of 100 people having radiation therapy, none die during the treatment, and 80 die by the end of five years. Which treatment would you advise him to choose?

**Question 4.** Pseudocertainty framing effect^b^

*Probabilistic certainty:* A certain disease is expected to afflict 20% of the population. Would you voluntarily receive a vaccine that protects half the people who receive it?

*Pseudocertainty:* There are two mutually exclusive and equiprobable virus strains of a certain disease, each of which is expected to afflict 10% of the population. Would you voluntarily receive a vaccine that gives complete protection against one strain and no protection against the other?

**Question 5.** Goal framing effect^c^

*Positive:* The doctor tells you that if you stop eating lipids, your body cholesterol content would be significantly reduced, and thus the possibility of suffering from cardiovascular disease would be greatly reduced. Would you continue to eat lipids?

*Negative:* The doctor tells you that if you continue to eat lipids, your body cholesterol content will significantly rise, and thus the possibility of suffering from cardiovascular disease would be greatly increased. Would you continue to eat lipids?

**Question 6.** Number-size framing effect^d^

*Framing 1:* Eye surgery may lead to two potential sequelae: one is a minor decline of visual acuity and the other is keratitis. Imagine you will take this kind of surgery and two doctors’ medical records revealed that: a) Doctor A: of 200 patients, 191 did not find a decline in postoperative visual acuity but 3 suffered from keratitis; b) Doctor B: of 200 patients, 198 did not find a decline in postoperative visual acuity but 10 suffered from keratitis. Which doctor you will choose?

*Framing 2:* Eye surgery may lead to two potential sequelae: one is a minor decline of visual acuity and the other is keratitis. Imagine you will take this kind of surgery and two doctors’ medical records revealed that: a) Doctor A: of 200 patients, 197 didn’t suffer from keratitis but in 9 visual acuity declined; b) Doctor B: of 200 patients, 190 didn’t suffer from keratitis but in 2 visual acuity declined. Which doctor you will choose?

**Question 7.** Attribute framing effect^e^

*Positive:* Among 100 patients taking one kind of medicine, 70 became better. How would you evaluate the drug effect?

*Negative:* Among 100 patients taking one kind of medicine, 30 patients didn’t become better. How would you evaluate the drug effect?

**Question 8.** Probabilistic formulation framing effect^f^

*Probability:*A vaccine that protects children from a deadly disease, has 0.001% risk of generating a permanent disability. Would you recommend vaccination?

*Raw data:* A vaccine that protects children from a deadly disease, can lead to permanent disability in one of every 100,000 children. Would you recommend vaccination?

^a^ Response was made in a Likert scale from "1" meaning "surely choose radiation therapy" to "6" meaning "surely choose surgery"

^b^ Response was made in a Likert scale from "1" meaning "almost certainly would not get vaccinated" to "6" meaning "almost certainly would get vaccinated"

^c^ Response was made in a Likert scale from "1" meaning "surely stop eating" to "6" meaning "surely continue eating"

^d^ Response was made in a Likert scale from "1" meaning "surely choose doctor A" to "6" meaning "surely choose doctor B"

^e^ Response was made in a Likert scale from "1" meaning "very bad" to "6" meaning "very good"

^f^ Response was made in a Likert scale from "1" meaning "surely would not recommend" to "6" meaning "surely would recommend"
